# Alteration of Blood Immune Biomarkers in MCI Patients with Different *APOE* Genotypes after Cognitive Training: A 1 Year Follow-Up Cohort Study

**DOI:** 10.3390/ijms241713395

**Published:** 2023-08-29

**Authors:** Olga Abramova, Yana Zorkina, Valeriya Ushakova, Dmitry Gryadunov, Anna Ikonnikova, Elena Fedoseeva, Marina Emelyanova, Aleksandra Ochneva, Irina Morozova, Konstantin Pavlov, Timur Syunyakov, Alisa Andryushchenko, Victor Savilov, Marat Kurmishev, Denis Andreuyk, Svetlana Shport, Olga Gurina, Vladimir Chekhonin, Georgy Kostyuk, Anna Morozova

**Affiliations:** 1 Mental-Health Clinic No. 1 Named after N.A. Alekseev, Zagorodnoe Highway 2, 115191 Moscow, Russia; abramova1128@gmail.com (O.A.); zorkina.ya@serbsky.ru (Y.Z.); ushakovavm@yandex.ru (V.U.); aleksochneva@yandex.ru (A.O.); irinashchelkanova@gmail.com (I.M.);; 2 Department of Basic and Applied Neurobiology, V. Serbsky Federal Medical Research Centre of Psychiatry and Narcology, Kropotkinsky per. 23, 119034 Moscow, Russia; 3 Biological Faculty, M.V. Lomonosov Moscow State University, 119991 Moscow, Russia; 4 Center for Precision Genome Editing and Genetic Technologies for Biomedicine, Engelhardt Institute of Molecular Biology, Russian Academy of Sciences, 119991 Moscow, Russia; 5 International Centre for Education and Research in Neuropsychiatry (ICERN), Samara State Medical University, 443016 Samara, Russia; 6 Department of Medical Nanobiotechnology, Pirogov Russian National Research Medical University, 117997 Moscow, Russia; 7 Department of Psychiatry, Federal State Budgetary Educational Institution of Higher Education “Moscow State University of Food Production”, Volokolamskoye Highway 11, 125080 Moscow, Russia

**Keywords:** cognitive dysfunction, dementia, MCI, immune biomarkers, cognitive training, *APOE*

## Abstract

Many studies aim to detect the early phase of dementia. One of the major ways to achieve this is to identify corresponding biomarkers, particularly immune blood biomarkers. The objective of this study was to identify such biomarkers in patients with mild cognitive impairment (MCI) in an experiment that included cognitive training. A group of patients with MCI diagnoses over the age of 65 participated in the study (n = 136). Measurements of cognitive functions (using the Mini-Mental State Examination scale and Montreal Cognitive Assessment) and determination of 27 serum biomarkers were performed twice: on the first visit and on the second visit, one year after the cognitive training. *APOE* genotypes were also determined. Concentrations of EGF (F = 17; *p* = 0.00007), Eotaxin (F = 7.17; *p* = 0.008), GRO (F = 13.42; *p* = 0.0004), IL-8 (F = 8.16; *p* = 0.005), MCP-1 (F = 13.46; *p* = 0.0001) and MDC (F = 5.93; *p* = 0.016) increased after the cognitive training in MCI patients. All these parameters except IL-8 demonstrated a weak correlation with other immune parameters and were poorly represented in the principal component analysis. Differences in concentrations of IP-10, FGF-2, TGFa and VEGF in patients with MCI were associated with *APOE* genotype. Therefore, the study identified several immune blood biomarkers that could potentially be associated with changes in cognitive function.

## 1. Introduction

Currently, more than 50 million people suffer from various types of dementia [1,2,3]. Alzheimer’s disease (AD) is the most common form of neurodegenerative dementia, affecting 5–6% of the population under 65 and up to 30% over 85 [4,5,6]. Therapy of such disorders is an important task due to significant economic costs and high mortality among the elderly population [3,5].

Many modern studies in the field of cognitive impairment therapy are aimed at identifying the early phase of the onset of dementia [7,8]. The pathological processes underlying such disorders, in particular AD, occur for many years before the onset of significant cognitive decline [7,9]. Their timely detection could potentially provide preventive therapeutic assistance. Mild cognitive impairment (MCI), in particular its amnestic form, is a prodromal stage of AD, being an intermediate phase between normal cognitive functioning in old age and dementia [10,11,12,13]. According to preliminary data, the likelihood of MCI progression in dementia of various kinds is 3–5 times higher than in individuals with a normal level of cognitive abilities [14,15]. In view of this, an important task is to identify the preclinical stage of dementia and search for appropriate biological markers [16,17]. An economic analysis has shown that treatment, expected to start in 2025, will delay the development of dementia by 5 years and reduce the incidence and associated economic costs by almost 40% over the next 25 years [18].

Multiple factors contribute to the development of cognitive impairment, such as high blood pressure, insulin resistance, metabolic syndrome, smoking, reduced physical activity, and plasma lipid levels [19]. Many studies have shown the role of changes in lipid composition in the etiology of dementia, in particular AD [20,21]. The association of AD with lipids was suggested by Alois Alzheimer after the discovery of the accumulation of lipid granules in glia [22]. The factor confirming the important role of lipids in the development of cognitive impairment is also the close relationship between the carriage of the epsilon 4 allele of the apolipoprotein gene (*APOE ɛ4*) and a high genetic risk of developing AD [23]. Inheritance of one or two copies of *APOE ɛ4* increases the risk of developing AD by about three and twelve times, respectively [24]. Other studies also confirm the association of lipid metabolism with dementia. It has been shown that changes in the level of total cholesterol can be a significant predisposition factor for cognitive disorders [25]. Moreover, high cholesterol levels may increase the risk of developing vascular dementia [26]. The literature suggests that the risk of developing MCI and dementia is associated with hypertriglyceridemia, high low-density lipoprotein (LDL) and low high-density lipoprotein (HDL) levels [27,28].

Neuroinflammation is one of the key mechanisms for the progression of neurodegenerative processes [29]. However, systemic inflammation also plays a key role in the pathogenesis of AD and other dementias [30]. Some hypotheses suggest that the inflammatory process may change the course of the disease, enhancing and exacerbating neurodegenerative processes [31,32]. Studies have shown that an increase in peripheral pro-inflammatory markers such as C-reactive protein (CRP), Interleukin-6 (IL-6) and soluble CD40 ligand (sCD40L) accompanies cognitive decline [33,34]. At the same time, an increase in the level of tumor necrosis factor receptors is associated with an increased risk of MCI progression to dementia [35]. Bawa et al. showed that a comprehensive assessment of neutrophil-associated inflammatory biomarkers such as neutrophil gelatinase-associated lipocalin (NGAL), myeloperoxidase (MPO), Interleukin-8 (IL-8), macrophage inflammatory protein 1β (MIP-1β), and tumor necrosis factor (TNF) can predict deterioration in functioning in patients with dementia over the course of a year [36]. A study by Oberlin et al. found that changes in peripheral inflammatory biomarkers are associated with increased amyloid deposition [37]. These data confirm the role of peripheral inflammation in the pathogenesis of cognitive impairment and point to the diagnostic role of inflammatory markers along with lipid metabolism parameters [38,39].

One of the main strategies for reducing the progression of MCI to dementia is cognitive training, which includes training of memory, attention and other mental functions [40,41,42]. The conduct of various neurocognitive rehabilitation programs produces a positive effect on the cognitive functions of patients with MCI [43,44]. The new paradigm of positive gerontology places emphasis on the ability to use mental resources in the third and fourth ages but not on disease development as a part of the normal aging process [45,46]. Modern approaches to the rehabilitation of cognitive deficits include the use of predictive models with multimodal markers [46,47]. That is important for medical workers for diagnosing and determining therapeutic and rehabilitation goals, as well as for non-medical specialists working with the problem of reducing cognitive competence at the stage of late ontogenesis [48,49]. Previously, we showed a positive effect of cognitive training that depended on the *APOE* genotype in patients with MCI [50].

The study of potential biomarkers of cognitive disorders is very important for the diagnosis and preventive therapy of dementia [51,52]. However, as single indicators have a low diagnostic value, in order to increase the efficiency of detection and therapy of pathologies, it is necessary to create entire diagnostic panels of biomarkers [53,54]. Equally important are long-term studies on the same patient’s cohorts, which allow for understanding the temporal course of the disease and identifying the main aspects of pathophysiological processes [55]. Previously, we conducted a comparative study of biomarkers of lipid metabolism and the immune system in patients with MCI and dementia, which showed changes in the levels of apolipoprotein A1 (Apo A1), HDL and some markers of the inflammatory process [56]. In the present work, we analyzed the trend of biomarker changes in patients with MCI one year after cognitive training to identify inflammatory blood markers that could potentially be associated with cognitive state. In addition, we conducted an analysis of the relationship between the level of inflammatory markers and the *APOE* genotype, since we assume that the genotype can determine the content of the biomarkers.

## 2. Results

### 2.1. General Characteristics of the Patient’s Cohort and the Assessment of Their Mental Status before and after the Cognitive Training

Complete data were collected from 136 people who applied to participate in the cognitive training program. The general characteristics of the study population are shown in Table 1.

Statistically significant differences in total Mini-Mental State Examination (MMSE) and Montreal Cognitive Assessment (MoCA) scores between the first and second visits were indicated. One year after the cognitive training, patients showed significantly higher scores on these scales, indicating improvement in cognitive function. However, the Hospital Anxiety and Depression Scale (HADS) score did not change, indicating that the level of depression and anxiety did not affect patients (Table 2).

### 2.2. The Measurement of Serum Markers before and after the Cognitive Training

Repeated Measures ANOVA showed that several immune blood parameters changed in MCI patients one year after the cognitive training (Table 3). Considering the false discovery rate (FDR) correction, significant differences were shown for five immune parameters. The concentrations of epidermal growth factor (EGF), Eotaxin-1 (Eotaxin), growth-regulated oncogene α (GRO), Interleukin-8 (IL-8) and monocyte chemoattractant protein 1 (MCP-1) increased after the cognitive training. Additionally, results were shown for the macrophage-derived chemokine (MDC) parameter; however, it did not pass the FDR.

**Figure 1:**
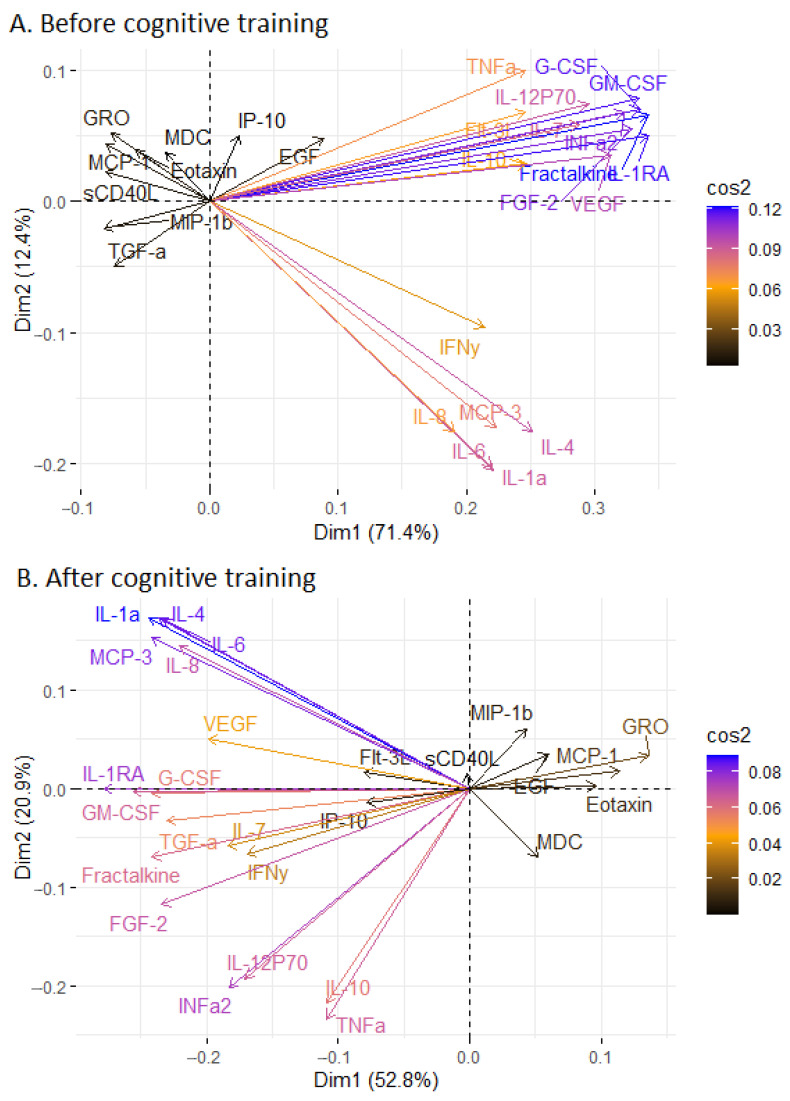
PCA of all blood immune parameters of MCI patients before and after cognitive training. (**A**) Biplots of the variables for the immune parameters before the cognitive training. (**B**) Biplots of the variables for the immune parameters after the cognitive training; Dim1: component 1; Dim2: component 2; cos2: the degree of the variable representation in each component. The ratio of cos2 changes is indicated by colored arrows. High cos2 values are colored blue and low cos2 values are colored black. Intermediate values are represented by a spectrum from violet (0.08) to brown (0.02) (from higher values to lower values, respectively).

After stratification according to the first and second visits, principal component analysis (PCA) revealed that the first principal component (Comp 1) and second principal component (Comp 2) explained most of the variance. In the first visit, Comp 1 and Comp 2 explained 71.4% and 12.4% of the variance, respectively (Figure 1A). In the second visit after one year, Comp 1 and Comp 2 explained 52.8% and 20.9% of the variance, respectively (Figure 1B). It should be noted that most of the changed immune parameters, which are described in Table 3, are poorly represented in Comp 1 and Comp 2 (with the exception of IL-8). The correlation analysis did not show a strong relationship between immune parameters and cognitive scale scores at both measurement points (Tables S1 and S2). 

**Figure 2:**
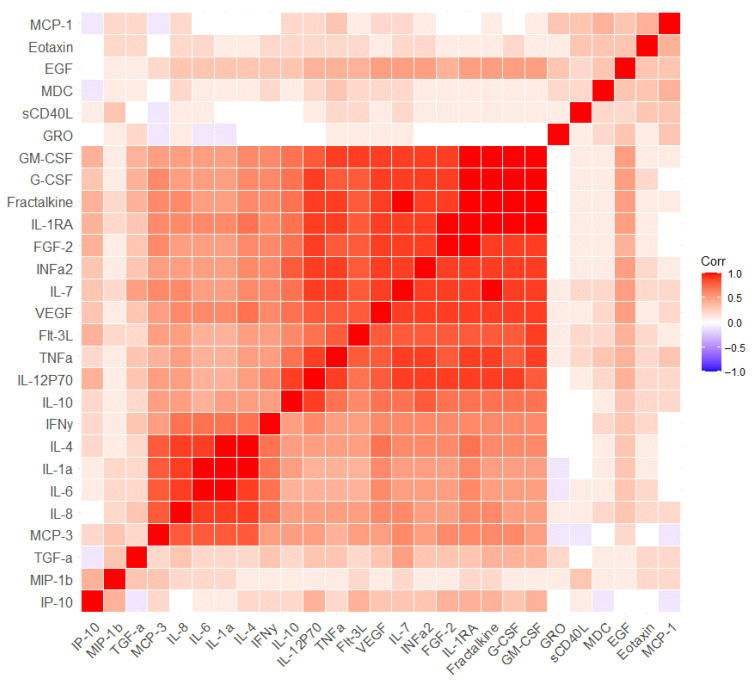
Correlation analysis of all blood immune parameters in MCI patients before cognitive training. Corr–Pearson correlation coefficient. Each cell represents a level of correlation between two serum biomarkers (horizontally and vertically, respectively). The Corr value is indicated in color. High Corr values are colored in red (positive correlation) and low Corr values are colored in blue (negative correlation). Intermediate values are represented by a spectrum from red to blue.

**Figure 3:**
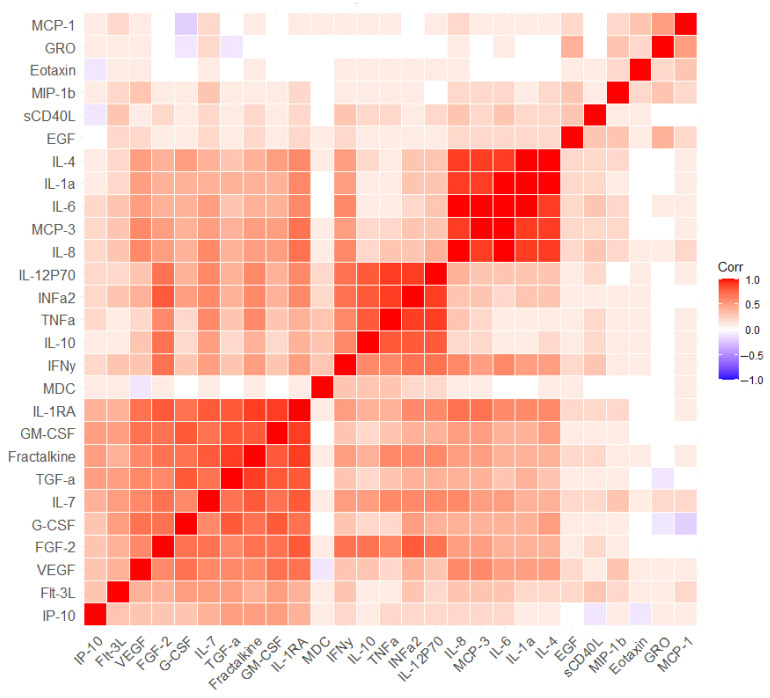
Correlation analysis of all blood immune parameters in MCI patients after cognitive training. Corr–Pearson correlation coefficient. Each cell represents a level of correlation between two serum biomarkers (horizontally and vertically, respectively). The Corr value is indicated in color. High Corr values are colored in red (positive correlation) and low Corr values are colored in blue (negative correlation). Intermediate values are represented by a spectrum from red to blue.

We performed a correlation analysis between blood immune parameters separately for the first and second visit points to identify clusters of interrelated parameters at each point (Figure 2 and Figure 3, Tables S1 and S2). It was shown that the first and second visits were characterized by a predominantly positive relationship between the concentrations of immune parameters. Only a few parameters demonstrated a negative relationship, but these relationships were weak. Before and after the visit, the same cluster of strongly positively related parameters can be identified, which was not affected by the cognitive training (Interleukin-4 (IL-4), Interleukin-1a (IL-1a), IL-6, IL-8 and monocyte chemotactic protein-3 (MCP-3)) (Figure 2 and Figure 3). For example, IL-8 was strongly correlated with IL-4 (r = 0.914, *p* < 0.001) and IL-6 (r = 0.945, *p* < 0.001) on the first visit point (Table S1). Approximately the same level of correlation remained a year later; IL-8 correlated with IL-4 (r = 0.918, *p* < 0.001) and IL-6 (r = 0.954, *p* < 0.001) (Table S2). Another larger cluster of immune parameters was also identified and could be observed on both visits. (granulocyte-macrophage colony-stimulating factor (GM-CSF), granulocyte colony-stimulating factor (G-CSF), Fractalkine, Interleukin-1RA (IL-1RA), fibroblast growth factor (FGF-2), Interferon α2 (INFa2), Interleukin-7 (IL-7), vascular endothelial growth factor (VEGF), fms-related tyrosine kinase 3 ligand (Flt-3L), TNFa, Interleukin-12P70 (IL-12P70), Interleukin-10 (IL-10) (Figure 2 and Figure 3). In particular, Fractalkine correlated with GM-CSF (r = 0.990, *p* < 0.001) on the first visit and on the second visit (r = 0.792, *p* < 0.001) (Tables S1 and S2). The only difference in the second cluster of immune markers was found in the transforming growth factor alpha (TGFα) parameter. In that case, TGFα correlated with GM-CSF (r = 0.847, *p* < 0.001), Fractalkine (r = 0.857, *p* < 0.001) and IL-1RA (r = 0.755, *p* < 0.001) on the second visit, while no associations were detected on the first visit (r = 0.391 for GM-CSF, r = 0.354 for Fractalkine, r = 0.326 for IL-1RA) (Tables S1 and S2). It was included in a large cluster at the second visit and was also well represented in the PCA components of the second visit, which was not observed for the first visit (Figure 1, Figure 2 and Figure 3). Separately, it is necessary to highlight another cluster of immune markers that are weakly correlated with others and which are poorly represented in the PCA for both visits. This cluster included sCD40L, EGF, Eotaxin, GRO, MCP-1 and MDC (Figure 1, Figure 2 and Figure 3). Notably, all these measures except sCD40L changed in MCI patients after the cognitive training (Table 3).

**Figure 4:**
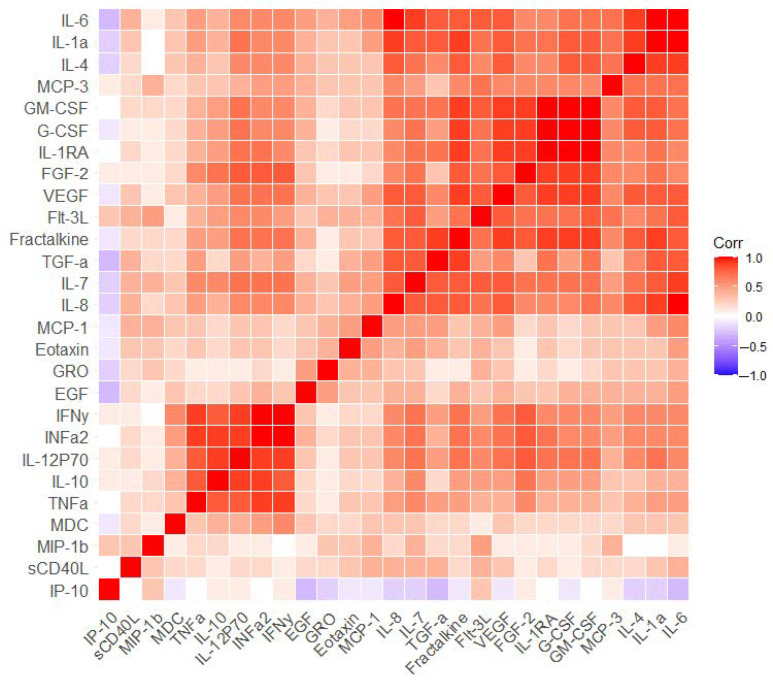
Correlation analysis of differences in immune parameter concentrations between the first and second visits in MCI patients. Corr–Pearson correlation coefficient. Each cell represents a level of correlation between two serum biomarkers (horizontally and vertically, respectively). The Corr value is indicated in color. High Corr values are colored in red (positive correlation) and low Corr values are colored in blue (negative correlation). Intermediate values are represented by a spectrum from red to blue.

We used another approach to evaluate the correlation between blood immune parameters. We determined the differences between the first and second visits for each parameter concentration and assessed the correlation between the differences. The results of this analysis are shown in Figure 4 and Table S3. The analysis indicated the presence of three clusters of significantly interconnected blood immune parameters. First cluster: Interferon γ (INFy), INFa2, IL-12P70, IL-10, TNFa; second cluster: GM-CSF, G-CSF, IL-1RA, FGF-2, VEGF, Fractalkine; and third cluster: IL-6, IL-1a, IL-4, IL-8. For example, INFα2 correlated with INFy (r = 0.967, *p* < 0.001), IL-10 (r = 0.855, *p* < 0.001), and IL-12P70 (r = 0.900, *p* < 0.001); FGF-2 correlated with G-CSF (r = 0.873, *p* < 0.001), GM-CSF (r = 0.853, *p* < 0.001), and VEGF (r = 0.807, *p* < 0.001) (Table S3). This analysis allowed us to identify groups of immune markers that were potentially associated with changes in MCI patients under the influence of cognitive training.

### 2.3. The Effect of APOE on Cognitive Scale Values and Associations with Serum Biomarkers

**Figure 5:**
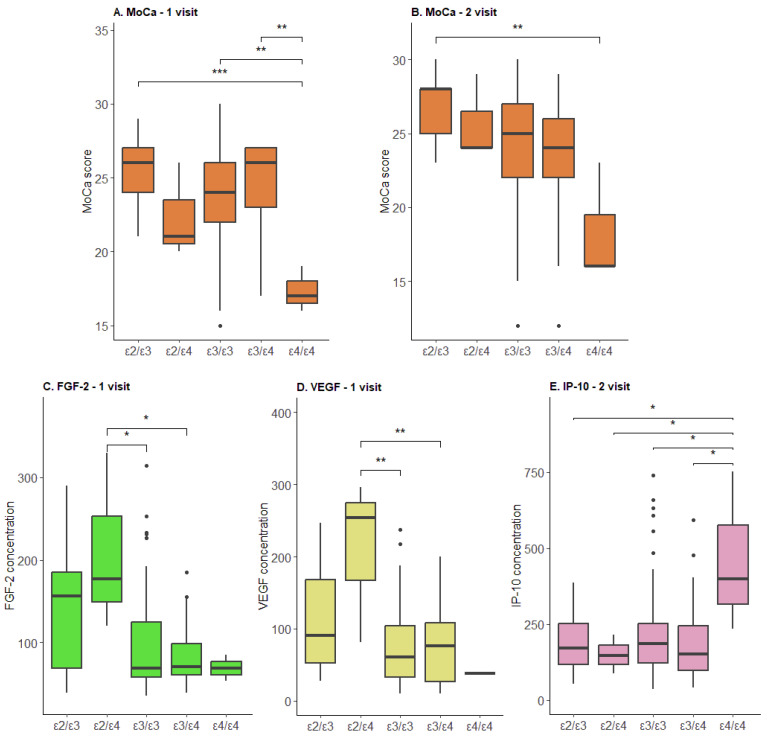
Cognitive scale parameters and concentration of immune biomarkers in the blood of patients with different *APOE* variants. (**A**) MoCA scale scores on the first visit; (**B**) MoCA scale scores on the second visit; (**C**) FGF-2 concentration on the first visit; (**D**) VEGF concentration on the first visit; and (**E**) IP-10 concentration on the second visit. *—*p* < 0.05; **—*p* < 0.01; ***—*p* < 0.001.

We evaluated the relationship between the concentration of immune parameters before and after cognitive training in patients with different *APOE* genotypes. Detailed results are presented in Table S4. We indicated that the *ε4/ε4* genotype had a significantly lower MoCA score at the first visit (Figure 5A) and at the second visit (Figure 5B). During the first visit, differences were detected in the blood parameters of FGF-2 and VEGF: the *ε3/ε3* and *ε3/ε4* genotypes had a significantly lower concentration compared to the *ε2/ε4* (Figure 5C,D) genotype. After the cognitive training, significant differences were shown only for the interferon γ-induced protein 10 kDa (IP-10) parameter; the *ε4/ε4* genotype demonstrated a higher concentration compared to all other genotypes (Figure 5E).

## 3. Discussion

In this study, we conducted long-term research on cognitive state and immune biomarkers alterations in MCI patients after cognitive training to identify possible indicators of cognitive changes.

Cognitive training is a rehabilitation program conducted in our hospital [57,58]. According to previous studies, such rehabilitation reduces the severity of cognitive manifestations and their progressive deterioration [50,57,58]. The goal of our neurocognitive rehabilitation strategy is to improve the quality of life of elderly citizens, increase the period of active longevity, restore cognitive deficits and prevent dementia. An interdisciplinary team approach involving psychiatrists, psychologists, medical professionals and social workers implemented a comprehensive treatment and rehabilitation program. Multidisciplinary training includes lifestyle changes, as it has been shown that this type of training is the most effective [44]. We have previously demonstrated a positive effect of cognitive training on cognitive functioning in MCI patients [50], and it is expected that it may produce an effect on some physiological and biochemical indicators of cognitive decline. Interestingly, cognitive training affected only cognitive test scores but did not influence depression or anxiety scores.

The current study showed that cognitive training also affected the concentrations of some immune parameters in the blood of patients with MCI. Below, we will discuss some of them in more detail.

EGF is an epidermal growth factor [59,60]. An alteration of this factor has been detected in AD and MCI patients [61,62]. In AD and MCI, low baseline plasma EGF levels predicted poorer long-term cognitive outcomes [61,63]. Other authors have indicated elevated plasma EGF concentrations in MCI [63] and AD patients [64,65]. A significant decrease in EGF levels in platelets in AD patients has also been demonstrated [66]. It was shown that EGF prevents amyloid-beta (Aβ)-induced brain endothelial cell damage in vitro [66]. In rodents, EGF prevented cognitive decline and was associated with a reduction of microhemorrhages but not with changes in Aβ levels [67].

The chemokine eotaxin-1 is a causal factor in cognitive decline during aging [68,69,70]. C-C chemokine receptor type 3 (CCR3), the receptor for eotaxin-1, is expressed by hippocampal neurons [68,71]. The treatment of primary hippocampal neuronal cultures with eotaxin-1 resulted in activation of cyclin-dependent kinase-5 and glycogen synthase-3β, which is associated with increased tau phosphorylation at several sites [69]. Eotaxin-1 treatment also induced Aβ formation and the loss of dendritic outgrowth in hippocampal neuron cultures [68]. Eotaxin-1 levels and measured levels of total tau were associated with amnestic MCI status in an African American cohort [72]. It was also shown that plasma eotaxin-1 levels correlated with the age of onset of AD [73,74]. In addition, elevated plasma eotaxin-1 levels have been associated with accelerated long-term forgetting [75,76]. 

MCP-1 is a chemokine of glial origin expressed by activated microglia [77,78]. It mediates neuroinflammation and may regulate memory performance in the elderly [77,78]. In MCI and AD, an interaction between MCP-1 and eotaxin-1 was observed, i.e., unfavorable associations with memory were observed when both chemokines were elevated. These associations remained significant after *APOE* genotype carriage [79]. Postmortem studies indicated increased levels of MCP-1 and Interleukin-1β (IL-1β) in parietal cortex and a trend toward increased levels of IL-1β and MCP-1 in frontal cortex in older adults with amyloid beta deposition compared to age-matched individuals without amyloid [80]. Four years of follow-up research demonstrated that baseline plasma MCP-1 levels are associated with a longitudinal decline in general cognitive and episodic memory scores in older adults. The most pronounced association of MCP-1 with cognitive decline was detected in individuals with amyloid plaques, defined by plasma Aβ42/40 levels [77].

Patients with AD had higher plasma MCP-1 levels compared to MCI patients and controls, with the highest levels shown in severe AD patients [77,81]. Baseline MCP-1 levels correlated significantly with MMSE changes [81,82]. MCP-1 levels also increased in urine, and these changes correlated with age [83]. Moreover, MCP-1 levels were significantly higher in patients with AD and amnestic MCI than in cognitively normal patients [83]. No association with cognitive impairment was described for GRO MDC markers. 

IL-8 is also associated with cognitive impairment, especially disturbances in attention, executive function, and visual-spatial function, suggesting a role of neuroinflammation in cognitive impairment [84]. Plasma IL-8 levels were lower in patients with MCI and AD compared to the normal control group [85,86]. A post-mortem study revealed decreased IL-8 concentrations in all dementia groups compared to the non-dementia population. In particular, IL-8 levels were significantly lower in patients with dementia compared to those without dementia in all regions [87].

IL-8 was also associated with the accumulation of pathological proteins characteristic of AD [88,89]. Lower levels of Aβ40 and Aβ42 and higher levels of IL-8 were associated with more severe cognitive decline [88,89,90]. Positron emission tomography (PET) research indicated negative associations of Aβ with cerebrospinal fluid (CSF) IL-8 levels in the areas where early Aβ accumulation occurs (in the lateral and medial frontal lobes) [91]. Negative associations of tau with IL-8 levels in CSF were also observed, predominantly in the areas where early accumulation of tau occurs (in the medial temporal lobe) [91]. Further analysis detected significant correlations between *APOE ɛ4* status and IL-8 in CSF and its influence on Aβ and tau PET levels in brain regions [91]. Other research has shown that IL-8 and MCP-1 exposure induce tau phosphorylation in human neuronal cells [92]. Higher baseline levels of IL-8 were associated with improved memory over time against a background of lower levels of p-tau and the p-tau/Aβ-42 ratio in CSF [92]. Higher levels of IL-6 in CSF were also associated with less noticeable changes in CSF p-tau over time [92]. These results are consistent with the hypothesis that increased brain IL-6 and IL-8 levels may play a neuroprotective role in cognitively healthy older adults with less pronounced AD pathology [93]. Correlation analysis revealed some parameters that correlated with each other more strongly, such as IL-8 or fractalkine [93]. Although it itself did not increase 1 year after the cognitive training, its changes correlated with those of the other 13 immune markers [93]. 

Thus, some inflammatory factors may play a key role in the development of neuroinflammation during cognitive impairment [73,77,81,94,95]. Further studies should be devoted to the investigation of the relationship between neuroinflammatory parameters and the identification of pathogenetic pathways influencing the development of cognitive impairment [94,95]. 

Inflammation is an important factor that can induce neurodegeneration. Inflammatory processes are observed in brain tissues from AD patients [96,97]. The presence of morphologically active microglia and astrocytes, elevated extracellular complement factors, cytokines and other inflammatory proteins, and elevated levels of inflammatory proteins in the brain and peripheral tissues in AD patients indicates the development of inflammation [93]. Multiple pieces of evidence demonstrate the critical role of *APOE* at the interface of inflammation and neurodegeneration through glial-mediated mechanisms [90]. *APOE* is considered a major carrier of lipid apolipoproteins and cholesterol in the central nervous system [90]. Many studies have focused on the research of liquor biomarkers that are associated with *APOE*, including such parameters as Aβ1–42 concentrations and the Aβ42/Aβ40 ratio [93]. Some studies have also shown the effect of *APOE* genotype on the concentration of biomarkers in the blood, such as melatonin [98], lipoproteins [99,100], triglyceride [101], anion gap, bicarbonate, albumin and glucose [102]. A number of studies have shown the effect of *APOE* genotype on inflammatory blood parameters, foremost C-reactive protein. Thus, elevated levels of C-reactive protein were associated with a reduced risk of longitudinal cognitive decline in older adults. However, this was observed only in those who did not carry the *APOE ɛ4* allele [103]. The study of Taiwanese older adults demonstrated that *APOE ɛ4* carriers were less likely to have elevated CRP levels compared to the non-carriers [104]. *APOE ɛ2* carriers exhibited the highest levels of CRP, followed by *APOE ɛ3* and *APOE ɛ4* [105]. 

Our study simultaneously tested a panel of 27 immune markers and showed that IP-10, FGF-2, TGFa and VEGF concentrations in MCI patients were associated with *APOE* genotype, suggesting their association with cognitive decline in the elderly. The findings support several other studies. For example, VEGF has previously been shown to be elevated in sporadic early- and late-stage AD compared to cognitively normal older adults and associated with cognitive ability and gray matter volumes [106]. High levels of IP-10 in plasma have been associated with poor cognitive test performance in patients with Parkinson’s disease [107]. Moderately to strongly negative correlations between social and cognitive functioning and serum TNFa and serum and urine IP-10 have been observed in patients with multiple sclerosis and healthy subjects [108]. IP-10 concentrations in CSF were significantly elevated in patients with MCI and mild AD but not in patients with severe AD. However, a significant positive correlation between MMSE score and IP-10 concentration in CSF was observed in patients with AD [109]. 

In our study, we also evaluated the correlations between different immune serum markers. This allowed us to identify a number of biomarkers associated with each other. For some of them, correlations were detected only on the second visit, one year after the start of the research. In particular, this pattern was observed for TGFα, which may indirectly indicate its association with cognitive functioning. It is also interesting to note that some immune biomarkers were weakly correlated with others, while their changes were statistically significant after the cognitive training (EGF, Eotaxin, GRO, MCP-1 and MDC). It can indicate their specific role in the therapeutic effect of cognitive training.

Nevertheless, despite the identified changes in immune markers, our study has some limitations that should be considered during future research. First, the main limitation of our study is the absence of a healthy control group, including individuals of similar age without signs of cognitive decline. It is important to measure serum biomarkers in healthy volunteers at the same time points before and after the cognitive training to confirm the obtained results and to assess long-term changes in serum immune parameters after the cognitive training more objectively.

Thus, in our study, we showed the alteration of some immune blood parameters concentrations in MCI patients after cognitive training (EGF, Eotaxin, GRO, IL-8, MCP-1 and MDC). The concentrations of other immune markers were associated with the *APOE* genotype (IP-10, FGF-2, TGFa and VEGF). In this view, we hypothesized that these alterations could play a part in cognitive function changes. As MCI often represents an early stage of dementia, these biomarkers could potentially act as indicators of cognitive impairment, allowing them to be used for the early diagnosis of cognitive decline. Such a diagnosis is important in the context of dementia prevention and preventive therapy. One of the possible methods of early-stage dementia therapy is cognitive training, as described in our study, which has been shown to be effective.

## 4. Materials and Methods

### 4.1. Patients

The study was conducted according to the guidelines of the Declaration of Helsinki. The procedures involving experiments on human subjects were performed in accordance with the ethical standards of Protocol No. 5, dated 20 September 2020, of the Ethics Committee of the Research Clinical Institute named after L.I. Sverzhevsky of the Moscow Healthcare Department.

The study included individuals observed at the Mental-Health Clinic No. 1, named after N.A. Alekseev, of the Moscow Healthcare Department from September 2020 to February 2022. The first point of observation was at the end of 2020 (15 September 2020 for the first subject, and the first follow-up took place on 7 October 2021).

A group of subjects with an MCI diagnosis over the age of 65 participated in the study, particularly individuals who requested the “Memory Clinic” of Mental-Health Clinic No. 1, named after N.A. Alekseev of the Moscow Healthcare Department, with subjective cognitive decline (n = 136). All diagnoses were established based on the results of regular interdisciplinary consultations involving neurologists, neuropsychologists, and psychiatrists. The diagnosis status was determined in accordance with the International Classification of Diseases (ICD-10). All participants underwent standardized neurological examinations and neuropsychological testing. The MMSE questionnaire was used to assess cognitive function. A score of 24 or below was considered dementia. These patients received cognitive training. The study included individuals with the following symptoms: complaints of forgetfulness, lack of attention and concentration (for example, when talking or reading a book), episodic difficulties in spatial orientation (for example, finding their way home), difficulties in phrasing and character, decreased professional and social productivity, impaired motor skills (writing, drawing) and problems in everyday routine (paying bills, shopping). 

On the first visit, the concentration of serum biomarkers (EGF, FGF-2, Eotaxin-1 (Eotaxin), TGFa, G-CSF, Fractalkine, INFa2, GRO, IL-10, MCP-3, MDC, sCD40L, IL-1Ra, IL-1a, IL-4, IL-6, IL-7, IL-8, IP-10, MCP-1, MIP-1β, TNFa, VEGF, IL-12P70, Flt-3L, GM-CSF) and *APOE* genotype were determined. At the first and second follow-up points, tests were performed using the MMSE scale and MoCA. The depression level was assessed with HADS. The patients then underwent cognitive training. The second visit included a reassessment of cognitive functions using the same scales and a re-measurement of serum immune parameters one year after the cognitive training.

Subjects were excluded due to any of the following: dementia; psychiatric illness; positive family histories (first-degree relatives) of psychiatric illness; substance abuse; or severe somatic diseases.

### 4.2. Neurocognitive Training

As part of the federal project “The Older Generation” and the regional program “Active Longevity”, in 2016 Moscow organized a network of “Memory Clinics”, consisting of clinical and rehabilitation units for daycare patients. An interdisciplinary team approach involving psychiatrists, psychologists, medical professionals and social workers was used to implement a comprehensive medical and rehabilitation program.

Fast recovery of individuals with cognitive decline after the medical and rehabilitation program and restoration of all components of the higher mental functions were shown; an adaptation of the program with reference to the conditions of the coronavirus pandemic did not demand increased duration or a cardinal change in the form of training. 

In order to determine the indications for the neurocognitive rehabilitation program, a psychiatrist evaluated the applicant using screening clinical scales and tests such as the Brief Mental Status Examination (MMSE) scale, the Clock Drawing Test (CDT), the HADS and the Modified Hachinski Ischemic Score (MHIS) [58].

Contraindications for the neurocognitive rehabilitation program were determined by the doctor at the pre-hospital examination and included: somatic diseases in the phase of exacerbation; pelvic organ dysfunction syndrome; pronounced depressive and anxiety-depressive disorders (HADS-A and HADS-D subscale scores more than 10 points); requiring specialized therapy; history of endogenous pathology; severe forms of cognitive disorders; episyndromes and absence of indications for cognitive rehabilitation.

The duration of the rehabilitation cycle was 6 weeks and 96 h of sessions, including cognitive training with a clear principle of progressive complexity and repeated practice of structured tasks to improve certain cognitive functions. Rehabilitation was conducted in a group format. Groups of 8–9 people were formed, taking into account age and cognitive similarity [58].

From week 2 to week 5, patients were included in the process of neurocognitive rehabilitation and psychosocial therapy every day (from 9.00 to 15.00). The training of the neurocognitive rehabilitation program for patients had a flexible structure and the possibility of changing the complexity depending on the status of the group. Cognitive trainings were combined; each type of training was indicated based on its purpose:

- CT-1 Correction of thinking and imagination (programming, regulation and control of complex activities);
- CT-2 Correction of amnestic activity and memory disorders;
- CT-3 Correction of attention and perception;
- CT-4 Art therapy for patients with mild cognitive decline;
- Cognitive warm-up—correction of praxis and gnosis.

Each training session consisted of structured and standardized sessions. In the presence of aphasia (motor, sensory, acoustic-amnestic and semantic) and mild dysarthria, the rehabilitation program included a speech therapy module and corresponding exercises depending on the specifics of the speech disorders. The speech therapy module was conducted by speech therapists.

In the morning hours, the participants were engaged in a set of therapeutic physical exercises—“adaptive physical training”. An instructor diagnosed the patient using D. Barthel’s “Daily Living Scale” (“The Barthel Index”) to assess the level of everyday activity and, based on the patient’s scores, established a regimen of adaptive physical training, depending on the patient’s age, physical condition, diseases and injuries: training mode (85–100 points), sparing regime (70–85 points).

Psychotherapeutic cognitive training was adapted for elderly and senile patients with cognitive decline, including methods of cognitive-behavioral, body-oriented, existential psychotherapy, creative expression therapy, neuromuscular relaxation and psychodrama, and was conducted daily with the patients of the clinic. The task of psychotherapeutic sessions was to reduce the level of destructive emotional states (fear, anxiety, anger and resentment) and to develop the skill of adequate acceptance of discomforting emotional experiences.

Drug therapy, if necessary, included nootropics, antioxidants, and vitamin therapy. In the presence of sub-anxiety and sub-depressive symptoms, patients were prescribed appropriate medications in minimally sufficient doses. 

At the final stage of the program (6th week), a repeated medical and psychological examination of the cognitive function state was conducted. A medical psychologist conducted testing on the MoCA, “10 words Luria” and SF-36 scales. Medical psychiatrists conducted testing on the MMSE, HADS and CDT scales. The key outcomes of psychosocial therapy and neurocognitive rehabilitation for patients with MCI were improvement of cognitive function, restoration of cognitive skills, improvement of social functioning and quality of life.

Further monitoring of patients who have completed the program was carried out by post-hospital monitoring (“post-hospital follow-up”). During the final stage, patients received appropriate methodological materials for individual work. Monitoring of task fulfillment was conducted six months later. The use of such a monitoring system allows for assessing the patient’s cognitive functions and determining the possible date of a repeat program cycle, if necessary.

### 4.3. Cognitive Impairment Scales

The MoCA scale and MMSE scale were used for cognitive impairment estimation. HADS was used for the assessment of depression and anxiety levels.

The MoCA is widely used to assess the cognitive functions of patients with various diseases [110,111]. Results are scored on a scale between 0 and 30, with a score of 26 or higher considered normal.

The MMSE is a short 30-item questionnaire widely used to assess and screen for cognitive impairment, including dementia, and to assess the dynamics of cognitive function against the background of ongoing therapy [8,112].

HADS is a summary measure of generalized symptoms of anxiety and fear. The goal of HADS is to screen for clinically significant symptoms of anxiety and depression in patients. HADS includes specific measures that determine general anxiety, including tension, anxiety, fear, panic, difficulty relaxing and restlessness [113,114].

### 4.4. Serum Sample Collection

The immunological parameters were determined in the serum. Blood from the cubital vein for the lipid analysis was sampled on an empty stomach until 9 a.m. Serum was separated immediately after blood sampling by centrifugation (3000 rpm for 10 min) at 4 °C and stored at −80 °C until the analysis.

### 4.5. APOE Genotyping

*APOE* genotyping was performed as described previously [50]. 

### 4.6. Multiplex Assay for Cytokine and Chemokine Measurement

The commercially available MILLIPLEX MAP Kit Human Cytokine/Chemokine Magnetic Bead Panel (Millipore, Billerica, MA, USA) was used as per the manufacturer’s instructions to measure serum immunological parameters. The following biomarkers were detected in patients’ serum samples: EGF, FDF-2, Eotaxin, TGFa, G-CSF, Fractalkine, INFa2, GRO, IL-10, MCP-3, MDC, sCD40L, IL-1Ra, IL-1a, IL-4, IL-6, IL-7, IL-8, IP-10, MCP-1, MIP-1β, TNFa, VEGF, IL-12P70, Flt-3L and GM-CSF.

### 4.7. Statistical Processing

The study population was characterized by means of descriptive statistics, providing means and standard errors (S.E.) for continuous variables and absolute and relative frequencies for categorical variables (Jamovi program, accessed on 1 September 2023). 

Repeated Measures ANOVA was used for evaluation of differences in HADS, MMSE and MoCA total scores and in immune parameters (Jamovi program, accessed on 1 September 2023). The data were presented as a mean ± SE. FDR correction was used for multiple comparisons. Differences were considered significant at *p* < 0.05. 

The data were normalized and used for PCA in RStudio (version 4.2.2). 

Pearson correlation was performed for the correlation analysis in the Jamovi program and RStudio. A r < −0.7 or r > 0.7 and *p* < 0.01 were considered statistically significant for the correlation analysis to account for multiple comparisons. For correlation analysis, the difference for each immune blood parameter between the first and second visits was determined using the formula: V1–V2, where V1 is the concentration in the i-th patient on the first visit and V2 is the concentration in the i-th patient on the second visit.

ANOVA was used to assess differences between ε-polymorphisms of the *APOE* gene (rs429358 and rs7412 variants) in HADS, MMSE and MoCA total scores and in immune parameters (Jamovi program, accessed on 1 September 2023; RStudio).

## Figures and Tables

**Table 1:** General characteristics of the study population.

Variable/Statistic	Categories	Value
Age, Mean (SE)		72.4 (0.64)
Sex, n (%)	Female	121 (89.0)
	Male	15 (11.0)
**Education and work**		
Vocational school, n (%)	No	66 (49.6)
	Yes	67 (50.4)
Not Magister, n (%)	No	126 (94.7)
	Yes	7 (5.3)
Magister, n (%)	No	46 (34.1)
	Yes	89 (65.9)
Years of University, n (%)	0	41 (30.6)
	1–3	5 (3.6)
	5	59 (44.0)
	6	24 (17.9)
	>7	5 (3.6)
Total years of school + vocational school, n (%)	8	1 (0.7)
	10	78 (57.8)
	11	7 (5.2)
	12	18 (13.3)
	13	20 (14.8)
	14	11 (8.1)
Years of university, mean (SE)		3.66 (0.22)
Total years of school + vocational school, mean (SE)		11.07 (0.12)
Type of work, n (%)	Intellectual	116 (86.6)
	Technical	18 (13.4)
**Family**		
Family, n (%)	Yes	95 (70.4)
	No	40 (29.6)
Number of children, n (%)	0	17 (12.7)
	1	54 (40.3)
	2	58 (43.3)
	3	5 (3.7)

**Table 2:** Evaluation of changes in scale scores in patients with MCI one year after cognitive training.

Scales	Baseline Examination		1 Year		F	df	*p*
	Mean	Std. Error of Mean	Mean	Std. Error of Mean			
MoCA ^1^	23.72	0.28	24.32	0.33	5.26	1	**0.02**
MMSE ^2^	26.80	0.16	28.29	0.17	68.3	1	**<0.001**
HADS ^3^	12.85	0.50	12.41	0.49	0.65	1	0.42

Repeated measures analysis was performed using repeated measures ANOVA. Significant *p*-values (<0.05) are highlighted in bold. ^1^—Mini-Mental State Examination; ^2^—Montreal Cognitive Assessment; ^3^—Hospital Anxiety and Depression Scale.

**Table 3:** Evaluation of changes in serum immune parameters in patients with MCI one year after cognitive training.

Immune Parameters	First Visit		Second Visit		F	*p*	FDR
	Mean	Std. Error of Mean	Mean	Std. Error of Mean			
**EGF**	**77.9**	**4.7**	106.2	6.9	17	**0.00007**	**0.002**
FGF 2	115.7	16.3	124	10.3	0.4	0.53	0.75
Eotaxin	135.3	7	161.6	10.4	7.17	**0.008**	**0.04**
TGFα	5.2	0.4	10.7	3.1	1.13	0.29	0.56
G-CSF	109.1	33.3	84.1	11.8	0.55	0.46	0.78
Fractalkine	240.7	92.3	248.6	49.8	0.01	0.91	0.98
INFα2	63.6	11.3	62.5	12.6	0.001	0.98	0.98
IFNγ	12.3	4.2	13.2	4.1	0.07	0.79	0.93
GRO	1409.1	77	1735.9	87.4	13.42	**0.0004**	**0.004**
IL-10	4.5	0.6	3.8	0.6	0.06	0.81	0.91
MCP-3	57.4	6.3	47.9	5.8	0.11	0.74	0.91
MDC	682	36.6	799.2	59	5.93	**0.016**	**0.07**
IL-12P70	7.5	2.3	7.6	2.2	0.003	0.96	1.00
sCD40L	2931.6	159.7	3095.4	79.5	1	0.32	0.58
IL-1RA	32.9	12.5	22.3	3.3	0.45	0.5	0.79
IL-1a	81.6	20.4	73.8	15.7	1.22	0.27	0.56
IL-4	369	78.6	322.8	69	0.34	0.56	0.76
IL-6	25	5.9	31.9	6.3	3.42	0.07	0.21
IL-7	15.6	1.7	17	1.2	1.49	0.23	0.52
IL-8	15.8	1.8	19.9	1.9	8.16	**0.005**	**0.03**
IP-10	269.1	20.1	230	15	3.5	0.06	0.20
MCP-1	543.8	27.9	640.8	22.8	13.46	**0.0003**	**0.004**
MIP-1 β	42.1	2.2	43.5	2.8	0.42	0.52	0.78
TNFa	16.9	1.7	21.3	3.2	2.84	0.09	0.24
VEGF	101.5	17.3	128.6	11.1	3.85	0.053	0.20
Flt-3L	43.0	5.6	42.1	2.9	1.85	0.18	0.45
GM-CSF	19.6	10.2	14.4	2.7	0.23	0.63	0.81

Repeated measures analysis was performed using repeated measures ANOVA. Significant *p*-values (<0.05) are highlighted in bold.

## Data Availability

Data are available on request.

## Supplementary Materials

The following supporting information can be downloaded at: https://www.mdpi.com/article/10.3390/ijms241713395/s1.

## Abbreviations

AD	Alzheimer’s disease
Apo A1	Apolipoprotein AI
*APOE*	Apolipoprotein
CCR3	C-C chemokine receptor type 3
CD14	Cluster of differentiation 14
CDT	Clock drawing test
CRP	C-reactive protein
CSF	Cerebrospinal fluid
EGF	Epidermal growth factor
FDF-2	Fibroblast growth factor
FDR	False discovery rate
Flt-3L	Fms-related tyrosine kinase 3 ligand
G-CSF	Granulocyte colony-stimulating factor
GM-CSF	Granulocyte-macrophage colony-stimulating factor
GRO	Growth-regulated oncogene α
HADS	Hospital Anxiety and Depression Scale
HDL	High-density lipoprotein
IL-10	Interleukin-10
IL-12P70	Interleukin-12P70
IL-1a	Interleukin-1a
IL-1Ra	Interleukin-1Ra
IL-4	Interleukin-4
IL-6	Interleukin-6
IL-7	Interleukin-7
IL-8	Interleukin-8
INFa2	Interferon α2
INFγ	Interferon γ
Interleukin-1β	Interleukin-1β
IP-10	Interferon γ-induced protein 10 kDa
LDL	Low-density lipoprotein
MCI	Mild cognitive impairment
MCP-1	Monocyte Chemoattractant Protein 1
MCP-3	Monocyte chemotactic protein-3
MDC	Macrophage-derived chemokine
MHIS	Modified Hachinski Ishemic Score
MIP-1β	Macrophage Inflammatory Protein 1β
MMSE	Mini-Mental State Examination
MoCA	Montreal Cognitive Assessment
MPO	Myeloperoxidase
NGAL	Neutrophil gelatinase-associated lipocalin
PCA	Principal component analysis
PET	Positron emission tomography
sCD40L	Soluble CD40 ligand
TGFa	Transforming Growth Factor Alpha
TNFα	Tumor necrosis factor α
VEGF	Vascular endothelial growth factor
