# Supplementary material for: Alteration of Blood Immune Biomarkers in MCI Patients with Different APOE Genotypes after Cognitive Training: A 1 Year Follow-Up Cohort Study
Source: Int J Mol Sci. 2023 Aug 29;24(17):13395. doi: 10.3390/ijms241713395 (PMC10488004; doi:10.3390/ijms241713395)
Supplement: Supplementary file 1 [file ijms-24-13395-s001.zip › ijms-2565905-supplementary.pdf]

Table S1. Correlation analysis of all blood immune parameters and scales of MCI patients before cognitive training. Significant relationships are highlighted in red (r<-0.7 or r>0.7, and p<0.01).

		MMSE	HADS	MoCa	EGF	FGF-2	Eotaxin	TGF-a	G-CSF	Flt-3L	GM-CSF	Fractalkine	INFa2	IFNy	GRO	IL-10	MCP-3	MDC	IL-12P70	sCD40L	IL-1RA	IL-1a	IL-4	IL-6	IL-7	IL-8	IP-10	MCP-1	MIP-1b	TNFa	VEGF
MMSE	Pearson r	—																													
	p-value	—																													
HADS	Pearson r	-0.049	—																												
	p-value	0.606	—																												
MoCa	Pearson r	0.479	-0.083	—																											
	p-value	< .001	0.379	—																											
EGF	Pearson r	0.188	-0.088	0.010	—																										
	p-value	0.042	0.352	0.912	—																										
FGF-2	Pearson r	-0.010	-0.085	-0.046	0.436	—																									
	p-value	0.922	0.409	0.652	< .001	—																									
Eotaxin	Pearson r	-0.050	-0.096	-0.135	0.281	0.069	—																								
	p-value	0.593	0.310	0.147	0.002	0.498	—																								
TGF-a	Pearson r	0.122	-0.048	-0.007	0.090	0.340	0.177	—																							
	p-value	0.270	0.672	0.951	0.415	0.002	0.108	—																							
G-CSF	Pearson r	0.014	-0.125	-0.073	0.519	**0.950**	0.162	0.390	—																						
	p-value	0.903	0.271	0.522	< .001	**< .001**	0.153	0.001	—																						
Flt-3L	Pearson r	-0.064	-0.170	-0.014	0.389	**0.821**	0.214	0.181	**0.836**	—																					
	p-value	0.571	0.135	0.902	< .001	**< .001**	0.056	0.115	**< .001**	—																					
GM-CSF	Pearson r	0.003	-0.133	-0.034	0.485	**0.949**	0.132	0.391	**0.984**	**0.857**	—																				
	p-value	0.977	0.248	0.765	< .001	**< .001**	0.249	< .001	**< .001**	**< .001**	—																				
Fractalkine	Pearson r	0.007	-0.119	-0.057	0.495	**0.949**	0.122	0.354	**0.983**	**0.842**	**0.990**	—																			
	p-value	0.949	0.264	0.595	< .001	**< .001**	0.252	0.002	**< .001**	**< .001**	**< .001**	—																			
INFa2	Pearson r	0.045	-0.121	-0.024	0.470	**0.919**	0.156	0.274	**0.921**	**0.762**	**0.913**	**0.940**	—																		
	p-value	0.677	0.264	0.825	< .001	**< .001**	0.146	0.020	**< .001**	**< .001**	**< .001**	**< .001**	—																		
IFNy	Pearson r	-0.050	0.021	-0.244	0.267	0.617	0.162	0.258	0.561	0.523	0.552	0.600	0.555	—																	
	p-value	0.633	0.844	0.018	0.010	< .001	0.122	0.022	< .001	< .001	< .001	< .001	< .001	—																	
GRO	Pearson r	0.269	-0.037	0.109	0.337	0.029	0.131	0.194	-0.002	0.074	0.000	0.006	-0.011	-0.038	—																
	p-value	0.004	0.695	0.244	< .001	0.778	0.160	0.076	0.985	0.517	0.997	0.953	0.921	0.719	—																
IL-10	Pearson r	0.114	-0.165	0.043	0.273	0.690	0.158	0.159	0.711	0.572	**0.702**	**0.712**	**0.759**	0.468	0.006	—															
	p-value	0.268	0.111	0.681	0.007	< .001	0.125	0.158	< .001	< .001	**< .001**	**< .001**	**< .001**	< .001	0.955	—															
MCP-3	Pearson r	0.007	0.019	-0.099	0.239	0.554	-0.024	0.179	0.565	0.478	0.542	0.569	0.511	0.539	-0.122	0.498	—														

	p-value	0.945	0.855	0.341	0.019	< .001	0.821	0.120	< .001	< .001	< .001	< .001	< .001	< .001	0.239	< .001	—														
MDC	Pearson r	0.063	-0.031	0.060	0.328	0.067	0.258	0.105	0.130	0.196	0.107	0.130	0.134	0.185	0.192	0.139	-0.015	—													
	p-value	0.500	0.740	0.519	< .001	0.507	0.005	0.341	0.252	0.080	0.350	0.222	0.214	0.075	0.038	0.177	0.888	—													
IL-12P70	Pearson r	0.029	-0.147	-0.021	0.402	**0.888**	0.184	0.307	**0.855**	**0.704**	**0.850**	**0.887**	**0.940**	0.611	0.006	**0.859**	0.497	0.173	—												
	p-value	0.792	0.191	0.854	< .001	**< .001**	0.098	0.008	**< .001**	**< .001**	**< .001**	**< .001**	**< .001**	< .001	0.958	**< .001**	< .001	0.118	—												
sCD40L	Pearson r	-0.101	-0.089	0.136	0.190	0.069	0.260	-0.000	0.056	0.213	0.118	0.099	0.098	0.042	0.123	-0.023	-0.091	0.189	0.140	—											
	p-value	0.326	0.389	0.183	0.061	0.545	0.010	1.000	0.658	0.099	0.354	0.411	0.418	0.723	0.226	0.841	0.432	0.063	0.273	—											
IL-1RA	Pearson r	-0.014	-0.107	-0.062	0.466	**0.956**	0.106	0.326	**0.981**	**0.847**	**0.991**	**0.992**	**0.917**	0.609	-0.003	**0.728**	0.585	0.103	**0.862**	0.102	—										
	p-value	0.891	0.314	0.558	< .001	**< .001**	0.315	0.003	**< .001**	**< .001**	**< .001**	**< .001**	**< .001**	< .001	0.979	**< .001**	< .001	0.326	**< .001**	0.388	—										
IL-1a	Pearson r	-0.026	0.044	-0.209	0.297	0.538	0.074	0.193	0.511	0.410	0.498	0.536	0.500	0.667	-0.102	0.487	0.841	0.077	0.444	0.024	0.579	—									
	p-value	0.818	0.700	0.063	0.008	< .001	0.516	0.118	< .001	< .001	< .001	< .001	< .001	< .001	0.372	< .001	< .001	0.499	< .001	0.849	< .001	—									
IL-4	Pearson r	0.016	-0.011	-0.157	0.323	0.613	0.071	0.191	0.604	0.494	0.601	0.631	0.571	0.664	-0.028	0.532	**0.848**	0.055	0.519	-0.011	0.668	**0.962**	—								
	p-value	0.874	0.914	0.123	0.001	< .001	0.488	0.084	< .001	< .001	< .001	< .001	< .001	< .001	0.782	< .001	**< .001**	0.590	< .001	0.923	< .001	**< .001**	—								
IL-6	Pearson r	-0.077	0.068	-0.261	0.321	0.521	0.161	0.216	0.510	0.442	0.504	0.539	0.486	**0.720**	-0.093	0.423	**0.839**	0.064	0.437	0.066	0.579	**0.977**	**0.948**	—							
	p-value	0.553	0.602	0.042	0.011	< .001	0.214	0.117	< .001	< .001	< .001	< .001	< .001	**< .001**	0.475	< .001	**< .001**	0.623	0.001	0.656	< .001	**< .001**	**< .001**	—							
IL-7	Pearson r	0.073	-0.143	-0.037	0.516	**0.889**	0.238	0.498	**0.945**	**0.800**	**0.945**	**0.951**	**0.904**	0.569	0.128	**0.745**	0.563	0.192	**0.878**	0.165	**0.937**	0.526	0.616	0.520	—						
	p-value	0.495	0.177	0.727	< .001	**< .001**	0.023	< .001	**< .001**	**< .001**	**< .001**	**< .001**	**< .001**	< .001	0.228	**< .001**	< .001	0.069	**< .001**	0.167	**< .001**	< .001	< .001	< .001	—						
IL-8	Pearson r	0.060	-0.057	-0.114	0.337	0.518	0.210	0.262	0.515	0.470	0.516	0.551	0.519	**0.733**	0.113	0.500	**0.785**	0.204	0.517	0.058	0.588	**0.928**	**0.914**	**0.945**	0.589	—					
	p-value	0.527	0.547	0.224	< .001	< .001	0.024	0.015	< .001	< .001	< .001	< .001	< .001	**< .001**	0.227	< .001	**< .001**	0.029	< .001	0.573	< .001	**< .001**	**< .001**	**< .001**	< .001	—					
IP-10	Pearson r	-0.144	-0.170	-0.051	0.038	0.446	-0.048	-0.119	0.312	0.409	0.441	0.391	0.319	0.199	-0.025	0.200	0.229	-0.117	0.431	0.130	0.423	0.108	0.194	0.092	0.345	0.035	—				
	p-value	0.121	0.068	0.581	0.686	< .001	0.607	0.280	0.005	< .001	< .001	< .001	0.002	0.056	0.791	0.050	0.025	0.207	< .001	0.199	< .001	0.339	0.055	0.475	< .001	0.710	—				
MCP-1	Pearson r	0.178	-0.216	0.110	0.259	0.008	0.431	0.190	0.045	0.064	0.071	0.064	0.125	0.037	0.304	0.208	-0.054	0.427	0.166	0.310	0.044	0.047	0.031	0.050	0.184	0.225	-0.088	—			
	p-value	0.055	0.020	0.234	0.005	0.936	< .001	0.081	0.689	0.570	0.535	0.545	0.243	0.723	< .001	0.041	0.600	< .001	0.134	0.002	0.677	0.679	0.761	0.700	0.078	0.015	0.343	—			
MIP-1b	Pearson r	-0.127	-0.114	-0.082	0.052	0.123	0.198	0.275	0.103	0.195	0.139	0.153	0.107	0.128	0.072	0.141	0.304	0.090	0.122	0.299	0.157	0.174	0.135	0.175	0.207	0.195	0.355	0.168	—		
	p-value	0.171	0.225	0.380	0.577	0.223	0.031	0.011	0.362	0.080	0.222	0.149	0.318	0.222	0.437	0.167	0.003	0.330	0.271	0.003	0.136	0.124	0.184	0.175	0.048	0.036	< .001	0.068	—		
TNFa	Pearson r	0.063	-0.210	0.112	0.412	**0.802**	0.174	0.280	**0.847**	**0.792**	**0.875**	**0.872**	**0.868**	0.457	0.149	**0.706**	0.391	0.280	**0.872**	0.160	**0.858**	0.366	0.482	0.378	**0.865**	0.473	0.160	0.261	0.126	—	
	p-value	0.529	0.035	0.261	< .001	**< .001**	0.079	0.010	**< .001**	**< .001**	**< .001**	**< .001**	**< .001**	< .001	0.132	**< .001**	< .001	0.004	**< .001**	0.146	**< .001**	0.001	< .001	0.004	**< .001**	< .001	0.104	0.007	0.203	—	
VEGF	Pearson r	0.008	-0.140	-0.036	0.514	**0.890**	0.120	0.259	**0.933**	**0.783**	**0.944**	**0.936**	**0.875**	0.581	0.103	0.672	0.540	0.207	**0.829**	0.101	**0.940**	0.560	0.663	0.591	**0.921**	0.616	0.258	0.153	0.086	**0.821**	—
	p-value	0.940	0.203	0.740	< .001	**< .001**	0.264	0.041	**< .001**	**< .001**	**< .001**	**< .001**	**< .001**	< .001	0.343	< .001	< .001	0.054	**< .001**	0.393	**< .001**	< .001	< .001	< .001	**< .001**	< .001	0.015	0.155	0.424	**< .001**	—

Table S2. Correlation analysis of all blood immune parameters and scales of MCI patients after cognitive training. Significant relationships are highlighted in red (r<-0.7 or r>0.7, and p<0.01).

		MoCA	MMSE	HADS	EGF	FGF-2	Eotaxin	TGF-a	G-CSF	Flt-3L	GM-CSF	Fractalkine	INFa2	IFNy	GRO	IL-10	MCP-3	MDC	IL-12P70	sCD40L	IL-1RA	IL-1a	IL-4	IL-6	IL-7	IL-8	IP-10	MCP-1	MIP-1b	TNFa	VEGF
MoCA	Pearson r	—																													
	p-value	—																													
MMSE	Pearson r	0.545	—																												
	p-value	< .001	—																												
HADS	Pearson r	0.014	-0.006	—																											
	p-value	0.869	0.947	—																											
EGF	Pearson r	-0.007	0.008	-0.089	—																										
	p-value	0.935	0.929	0.306	—																										
FGF-2	Pearson r	-0.016	-0.042	-0.150	0.131	—																									
	p-value	0.864	0.655	0.112	0.159	—																									
Eotaxin	Pearson r	-0.236	-0.323	-0.094	0.066	-0.021	—																								
	p-value	0.006	< .001	0.285	0.445	0.826	—																								
TGF-a	Pearson r	-0.037	0.083	-0.151	0.115	0.600	0.017	—																							
	p-value	0.685	0.362	0.095	0.202	< .001	0.848	—																							
G-CSF	Pearson r	-0.020	-0.008	-0.230	0.132	0.669	-0.008	0.779	—																						
	p-value	0.836	0.936	0.017	0.172	< .001	0.932	< .001	—																						
Flt-3L	Pearson r	-0.133	-0.045	-0.207	0.221	0.407	0.098	0.462	0.478	—																					
	p-value	0.147	0.625	0.024	0.015	< .001	0.287	< .001	< .001	—																					
GM-CSF	Pearson r	0.029	0.072	-0.179	0.108	0.692	0.017	**0.847**	**0.822**	0.461	—																				
	p-value	0.762	0.461	0.064	0.261	< .001	0.857	**< .001**	**< .001**	< .001	—																				
Fractalkine	Pearson r	-0.073	0.075	-0.189	0.188	**0.725**	0.069	**0.857**	0.688	0.381	**0.792**	—																			
	p-value	0.484	0.474	0.068	0.066	**< .001**	0.505	**< .001**	< .001	< .001	**< .001**	—																			
INFa2	Pearson r	-0.052	-0.005	-0.171	0.129	**0.799**	0.054	0.444	0.468	0.286	0.429	0.587	—																		
	p-value	0.622	0.958	0.103	0.214	**< .001**	0.607	< .001	< .001	0.008	< .001	< .001	—																		
IFNy	Pearson r	-0.260	-0.196	-0.015	0.199	0.697	0.062	0.280	0.347	0.290	0.251	0.466	0.661	—																	
	p-value	0.009	0.052	0.882	0.047	< .001	0.540	0.007	0.001	0.005	0.019	< .001	< .001	—																	
GRO	Pearson r	-0.097	-0.107	-0.090	0.382	0.016	0.192	-0.065	-0.092	0.099	-0.048	-0.030	-0.017	0.027	—																
	p-value	0.265	0.218	0.305	< .001	0.866	0.025	0.469	0.344	0.276	0.622	0.772	0.870	0.786	—																
IL-10	Pearson r	-0.018	-0.035	-0.061	0.103	0.663	0.064	0.186	0.220	0.112	0.241	0.431	**0.833**	0.601	0.035	—															
	p-value	0.839	0.691	0.493	0.239	< .001	0.470	0.041	0.024	0.228	0.013	< .001	**< .001**	< .001	0.687	—															
MCP-3	Pearson r	-0.119	-0.142	-0.051	0.197	0.490	0.009	0.393	0.425	0.277	0.499	0.503	0.331	0.487	0.030	0.227	—														
	p-value	0.187	0.116	0.579	0.027	< .001	0.924	< .001	< .001	0.003	< .001	< .001	0.001	< .001	0.737	0.011	—														

MDC	Pearson r	-0.068	-0.007	0.089	0.147	0.097	0.050	-0.015	-0.032	0.074	-0.033	0.054	0.195	0.253	0.019	0.256	0.066	—													
	p-value	0.433	0.933	0.309	0.088	0.298	0.568	0.868	0.744	0.420	0.736	0.604	0.060	0.011	0.829	0.003	0.461	—													
IL-12P70	Pearson r	-0.105	-0.028	-0.150	0.111	**0.730**	0.064	0.398	0.409	0.225	0.374	0.601	**0.904**	**0.709**	-0.001	**0.784**	0.332	0.213	—												
	p-value	0.264	0.770	0.112	0.235	**< .001**	0.492	< .001	< .001	0.020	< .001	< .001	**< .001**	**< .001**	0.990	**< .001**	< .001	0.022	—												
sCD40L	Pearson r	-0.091	-0.080	-0.022	0.322	0.152	0.086	0.123	0.081	0.271	0.052	0.200	0.117	0.339	0.019	0.200	0.162	0.038	0.191	—											
	p-value	0.294	0.359	0.802	< .001	0.102	0.321	0.171	0.404	0.003	0.593	0.050	0.261	< .001	0.830	0.021	0.070	0.663	0.040	—											
IL-1RA	Pearson r	-0.042	0.012	-0.116	0.185	**0.801**	0.031	**0.755**	**0.722**	0.439	**0.876**	**0.858**	0.583	0.531	0.002	0.443	**0.714**	0.052	0.550	0.192	—										
	p-value	0.641	0.890	0.193	0.035	**< .001**	0.727	**< .001**	**< .001**	< .001	**< .001**	**< .001**	< .001	< .001	0.979	< .001	**< .001**	0.559	< .001	0.029	—										
IL-1a	Pearson r	-0.164	-0.148	-0.046	0.151	0.426	-0.009	0.359	0.439	0.247	0.445	0.436	0.260	0.550	-0.026	0.147	**0.942**	0.028	0.311	0.200	0.646	—									
	p-value	0.085	0.121	0.630	0.111	< .001	0.921	< .001	< .001	0.013	< .001	< .001	0.019	< .001	0.786	0.125	**< .001**	0.766	0.002	0.035	< .001	—									
IL-4	Pearson r	-0.164	-0.139	-0.058	0.172	0.426	0.002	0.343	0.462	0.241	0.457	0.433	0.279	0.543	0.026	0.158	**0.923**	0.060	0.326	0.180	0.644	**0.964**	—								
	p-value	0.058	0.111	0.505	0.046	< .001	0.980	< .001	< .001	0.008	< .001	< .001	0.006	< .001	0.766	0.070	**< .001**	0.492	< .001	0.037	< .001	**< .001**	—								
IL-6	Pearson r	-0.308	-0.237	-0.071	0.199	0.378	-0.023	0.349	0.429	0.334	0.398	0.436	0.233	0.556	0.066	0.068	**0.959**	0.004	0.304	0.279	0.633	**0.976**	**0.949**	—							
	p-value	0.010	0.050	0.562	0.101	0.002	0.849	0.005	< .001	0.006	0.001	< .001	0.084	< .001	0.588	0.579	**< .001**	0.972	0.014	0.020	< .001	**< .001**	**< .001**	—							
IL-7	Pearson r	-0.117	-0.137	-0.149	0.234	**0.733**	0.130	0.683	0.632	0.455	**0.732**	**0.829**	0.622	0.503	0.177	0.501	0.514	0.113	0.622	0.159	**0.802**	0.426	0.432	0.453	—						
	p-value	0.199	0.133	0.104	0.010	**< .001**	0.155	< .001	< .001	< .001	**< .001**	**< .001**	< .001	< .001	0.051	< .001	< .001	0.216	< .001	0.080	**< .001**	< .001	< .001	< .001	—						
IL-8	Pearson r	-0.169	-0.177	-0.058	0.206	0.492	0.080	0.388	0.427	0.336	0.483	0.503	0.340	0.605	0.085	0.220	**0.912**	0.114	0.434	0.258	**0.707**	**0.926**	**0.918**	**0.954**	0.561	—					
	p-value	0.051	0.041	0.511	0.016	< .001	0.357	< .001	< .001	< .001	< .001	< .001	< .001	< .001	0.327	0.011	**< .001**	0.185	< .001	0.002	**< .001**	**< .001**	**< .001**	**< .001**	< .001	—					
IP-10	Pearson r	-0.076	-0.049	-0.086	-0.031	0.266	-0.066	0.464	0.332	0.322	0.510	0.493	0.192	0.154	0.087	0.078	0.169	0.066	0.156	-0.065	0.352	0.132	0.118	0.222	0.438	0.202	—				
	p-value	0.382	0.577	0.326	0.717	0.004	0.448	< .001	< .001	< .001	< .001	< .001	0.063	0.127	0.316	0.376	0.058	0.445	0.095	0.452	< .001	0.166	0.173	0.067	< .001	0.019	—				
MCP-1	Pearson r	-0.123	-0.178	-0.065	0.240	0.016	0.330	0.009	-0.151	0.199	0.005	0.101	0.043	0.064	0.453	0.063	0.098	0.139	0.133	0.007	0.080	0.054	0.064	0.135	0.229	0.186	0.076	—			
	p-value	0.154	0.040	0.458	0.005	0.864	< .001	0.918	0.119	0.028	0.956	0.326	0.683	0.526	< .001	0.471	0.275	0.106	0.155	0.935	0.364	0.568	0.459	0.268	0.011	0.030	0.377	—			
MIP-1b	Pearson r	-0.204	-0.238	-0.110	0.256	0.126	0.194	0.077	0.078	0.191	0.120	0.131	0.070	0.084	0.297	0.102	0.246	0.015	0.023	0.127	0.181	0.134	0.153	0.209	0.290	0.236	0.104	0.209	—		
	p-value	0.017	0.006	0.209	0.003	0.175	0.024	0.396	0.423	0.035	0.211	0.204	0.503	0.406	< .001	0.243	0.005	0.862	0.807	0.141	0.040	0.159	0.077	0.085	0.001	0.006	0.226	0.015	—		
TNFa	Pearson r	-0.061	0.023	-0.112	0.134	0.611	0.059	0.343	0.216	0.145	0.252	0.559	**0.893**	0.603	0.035	**0.805**	0.215	0.273	**0.894**	0.155	0.447	0.148	0.138	0.143	0.561	0.254	0.184	0.145	0.105	—	
	p-value	0.488	0.791	0.202	0.123	< .001	0.497	< .001	0.025	0.114	0.008	< .001	**< .001**	< .001	0.692	**< .001**	0.016	0.001	**< .001**	0.073	< .001	0.122	0.113	0.242	< .001	0.003	0.033	0.094	0.226	—	
VEGF	Pearson r	-0.077	-0.129	-0.092	0.153	0.582	0.075	0.628	0.655	0.373	0.699	0.584	0.371	0.336	0.068	0.256	0.577	-0.055	0.346	0.133	**0.717**	0.544	0.526	0.529	0.630	0.596	0.336	0.051	0.343	0.198	—
	p-value	0.449	0.207	0.368	0.131	< .001	0.462	< .001	< .001	< .001	< .001	< .001	0.001	0.003	0.505	0.011	< .001	0.588	< .001	0.190	**< .001**	< .001	< .001	< .001	< .001	< .001	< .001	0.613	< .001	0.051	—

Table S3. Correlation analysis of difference in concentrations of immune parameters between the first and second visits in MCI patients. Significant relationships are highlighted in red (r<-0.7 or r>0.7, and p<0.01).

		EGF	FGF-2	Eotaxin	TGF-a	G-CSF	Flt-3L	GM-CSF	Fractalkine	INFa2	IFNy	GRO	IL-10	MCP-3	MDC	IL-12P70	sCD40L	IL-1RA	IL-1a	IL-4	IL-6	IL-7	IL-8	IP-10	MCP-1	MIP-1b	TNFa	VEGF
EGF	Pearson r	—																										
	p-value	—																										
FGF-2	Pearson r	0.324	—																									
	p-value	0.002	—																									
Eotaxin	Pearson r	0.259	0.137	—																								
	p-value	0.004	0.203	—																								
TGF-a	Pearson r	0.199	0.293	0.387	—																							
	p-value	0.077	0.019	< .001	—																							
G-CSF	Pearson r	0.373	**0.873**	0.214	0.494	—																						
	p-value	0.001	**< .001**	0.078	< .001	—																						
Flt-3L	Pearson r	0.336	**0.734**	0.434	0.517	**0.738**	—																					
	p-value	0.004	**< .001**	< .001	< .001	**< .001**	—																					
GM-CSF	Pearson r	0.373	**0.853**	0.309	**0.744**	**0.974**	**0.816**	—																				
	p-value	0.002	**< .001**	0.013	**< .001**	**< .001**	**< .001**	—																				
Fractalkine	Pearson r	0.378	**0.807**	0.291	**0.901**	**0.894**	**0.736**	**0.927**	—																			
	p-value	0.002	**< .001**	0.018	**< .001**	**< .001**	**< .001**	**< .001**	—																			
INFa2	Pearson r	0.359	**0.818**	0.254	0.385	0.629	0.558	0.605	0.663	—																		
	p-value	0.003	**< .001**	0.038	0.004	< .001	< .001	< .001	< .001	—																		
IFNy	Pearson r	0.266	**0.811**	0.163	0.457	0.615	0.507	0.601	0.687	**0.967**	—																	
	p-value	0.034	**< .001**	0.202	< .001	< .001	< .001	< .001	< .001	**< .001**	—																	
GRO	Pearson r	0.507	0.128	0.369	0.068	0.145	0.401	0.185	0.129	0.180	0.132	—																
	p-value	< .001	0.234	< .001	0.553	0.228	< .001	0.139	0.295	0.139	0.294	—																
IL-10	Pearson r	0.219	0.660	0.245	0.222	0.457	0.462	0.470	0.525	**0.855**	**0.831**	0.130	—															
	p-value	0.033	< .001	0.017	0.054	< .001	< .001	< .001	< .001	**< .001**	**< .001**	0.206	—															
MCP-3	Pearson r	0.438	0.641	0.253	0.330	0.560	0.658	0.557	0.568	0.513	0.490	0.269	0.327	—														
	p-value	< .001	< .001	0.015	0.005	< .001	< .001	< .001	< .001	< .001	< .001	0.009	0.003	—														
MDC	Pearson r	0.288	0.225	0.181	0.150	0.200	0.131	0.178	0.210	0.455	0.592	0.223	0.415	0.219	—													
	p-value	0.001	0.034	0.038	0.186	0.096	0.274	0.159	0.087	< .001	< .001	0.010	< .001	0.034	—													
IL-12P70	Pearson r	0.336	**0.797**	0.297	0.506	**0.715**	0.581	0.680	**0.738**	**0.900**	**0.909**	0.147	**0.864**	0.440	0.438	—												
	p-value	0.005	**< .001**	0.015	< .001	**< .001**	< .001	< .001	**< .001**	**< .001**	**< .001**	0.232	**< .001**	< .001	< .001	—												
sCD40L	Pearson r	0.173	0.098	0.320	0.411	0.099	0.367	0.188	0.227	0.200	0.070	0.227	0.096	0.179	0.179	0.159	—											
	p-value	0.082	0.415	< .001	0.001	0.463	0.005	0.173	0.098	0.148	0.617	0.015	0.404	0.124	0.056	0.265	—											
IL-1RA	Pearson r	0.345	**0.884**	0.258	0.699	**0.970**	**0.744**	**0.992**	**0.928**	0.653	0.649	0.166	0.491	0.588	0.197	**0.710**	0.198	—										

	p-value	< .001	**< .001**	0.015	< .001	**< .001**	**< .001**	**< .001**	**< .001**	< .001	< .001	0.118	< .001	< .001	0.062	**< .001**	0.095	—										
IL-1a	Pearson r	0.439	0.688	0.350	**0.812**	**0.795**	0.694	**0.831**	**0.889**	0.629	0.584	0.298	0.421	0.654	0.274	0.682	0.345	0.692	—									
	p-value	< .001	< .001	0.002	**< .001**	**< .001**	< .001	**< .001**	**< .001**	< .001	< .001	0.010	< .001	< .001	0.017	< .001	0.006	< .001	—									
IL-4	Pearson r	0.437	0.635	0.269	0.622	**0.768**	0.578	**0.782**	**0.843**	0.584	0.557	0.281	0.347	0.654	0.258	0.594	0.237	0.636	**0.950**	—								
	p-value	< .001	< .001	0.007	< .001	**< .001**	< .001	**< .001**	**< .001**	< .001	< .001	0.005	< .001	< .001	0.010	< .001	0.034	< .001	**< .001**	—								
IL-6	Pearson r	0.532	0.625	0.541	**0.829**	**0.731**	**0.766**	**0.738**	**0.849**	0.611	0.564	0.363	0.380	0.698	0.276	0.649	0.419	0.636	**0.972**	**0.943**	—							
	p-value	< .001	< .001	< .001	**< .001**	**< .001**	**< .001**	**< .001**	**< .001**	< .001	< .001	0.011	0.012	< .001	0.060	< .001	0.008	< .001	**< .001**	**< .001**	—							
IL-7	Pearson r	0.442	0.676	0.488	**0.766**	**0.704**	**0.779**	**0.747**	**0.831**	0.683	0.650	0.332	0.564	0.542	0.315	**0.727**	0.364	**0.725**	**0.823**	**0.715**	**0.865**	—						
	p-value	< .001	< .001	< .001	**< .001**	**< .001**	**< .001**	**< .001**	**< .001**	< .001	< .001	0.002	< .001	< .001	0.004	**< .001**	0.002	**< .001**	**< .001**	**< .001**	**< .001**	—						
IL-8	Pearson r	0.402	0.567	0.398	**0.800**	0.605	**0.716**	0.678	**0.762**	0.611	0.562	0.323	0.397	0.648	0.278	0.626	0.359	0.615	**0.924**	**0.848**	**0.964**	**0.847**	—					
	p-value	< .001	< .001	< .001	**< .001**	< .001	**< .001**	< .001	**< .001**	< .001	< .001	< .001	< .001	< .001	0.002	< .001	< .001	< .001	**< .001**	**< .001**	**< .001**	**< .001**	—					
IP-10	Pearson r	-0.332	0.149	-0.054	-0.315	-0.109	0.324	-0.040	-0.123	-0.008	0.089	-0.151	0.081	0.074	-0.096	0.066	-0.015	-0.005	-0.216	-0.170	-0.275	-0.154	-0.165	—				
	p-value	< .001	0.160	0.538	0.004	0.364	0.005	0.749	0.318	0.948	0.482	0.081	0.430	0.475	0.266	0.592	0.872	0.963	0.060	0.090	0.059	0.161	0.063	—				
MCP-1	Pearson r	0.289	0.151	0.482	0.535	0.160	0.389	0.311	0.329	0.301	0.177	0.432	0.249	0.334	0.334	0.311	0.393	0.279	0.484	0.345	0.561	0.506	0.535	-0.069	—			
	p-value	0.001	0.155	< .001	< .001	0.182	< .001	0.012	0.006	0.012	0.159	< .001	0.014	< .001	< .001	0.010	< .001	0.007	< .001	< .001	< .001	< .001	< .001	0.421	—			
MIP-1b	Pearson r	0.071	0.115	0.346	0.219	0.056	0.477	0.166	0.169	0.062	-0.006	0.343	0.219	0.407	0.091	0.130	0.342	0.122	0.034	-0.009	0.092	0.407	0.197	0.330	0.380	—		
	p-value	0.439	0.280	< .001	0.051	0.645	< .001	0.186	0.167	0.615	0.963	< .001	0.031	< .001	0.297	0.292	< .001	0.251	0.769	0.928	0.534	< .001	0.027	< .001	< .001	—		
TNFa	Pearson r	0.209	0.560	0.266	0.539	0.351	0.363	0.368	0.513	**0.902**	**0.891**	0.138	**0.791**	0.323	0.350	**0.827**	0.230	0.403	0.514	0.424	0.516	0.626	0.538	-0.037	0.310	0.220	—	
	p-value	0.033	< .001	0.005	< .001	0.003	0.002	0.003	< .001	**< .001**	**< .001**	0.150	**< .001**	0.002	< .001	**< .001**	0.027	< .001	< .001	< .001	< .001	< .001	< .001	0.696	< .001	0.020	—	
VEGF	Pearson r	0.391	**0.807**	0.286	0.610	**0.900**	**0.788**	**0.928**	**0.884**	0.653	0.663	0.260	0.471	0.641	0.318	0.692	0.264	**0.889**	**0.847**	**0.799**	**0.826**	**0.800**	**0.800**	-0.110	0.455	0.123	0.399	—
	p-value	< .001	**< .001**	0.012	< .001	**< .001**	**< .001**	**< .001**	**< .001**	< .001	< .001	0.023	< .001	< .001	0.005	< .001	0.036	**< .001**	**< .001**	**< .001**	**< .001**	**< .001**	**< .001**	0.342	< .001	0.287	< .001	—

Table S4. Evaluation of the relationship between the concentration of immune parameters before and after cognitive training in patients with different APOE genotypes. ANOVA was used for analysis. Significant differences in p-values are highlighted in bold.

Immune parameters / Scales	APOE genotypes			
	First visit		Second visit	
	F	*p*	*F*	*p*
EGF	0.60	0.66	1.87	0.12
FGF-2	3.83	**0.007**	0.73	0.57
Eotaxin	0.58	0.68	0.20	0.94
TGF-a	2.23	0.07	0.29	0.88
G-CSF	1.82	0.13	0.73	0.58
Fractalkine	0.88	0.48	1.07	0.37
INFa2	0.17	0.95	0.44	0.78
IFNy	0.30	0.87	0.40	0.81
GRO	0.33	0.86	1.03	0.39
IL-10	0.17	0.95	0.27	0.89
MCP-3	0.35	0.85	0.85	0.49
MDC	0.44	0.78	1.10	0.36
IL-12P70	0.25	0.91	0.34	0.85
sCD40L	0.38	0.82	0.31	0.87
IL-1RA	1.60	0.18	0.71	0.59
IL-1a	1.55	0.19	2.17	0.08
IL-4	1.16	0.33	0.53	0.71
IL-6	1.22	0.32	0.78	0.54
IL-7	0.25	0.91	0.78	0.54
IL-8	1.19	0.32	0.53	0.71
IP-10	0.15	0.96	2.93	**0.02**
MCP-1	0.35	0.84	2.08	0.09
MIP-1b	0.45	0.77	0.52	0.72
TNFa	0.46	0.76	0.44	0.78
VEGF	4.50	**0.002**	0.94	0.44
Flt-3L	1.12	0.35	0.38	0.82
GM-CSF	0.94	0.42	0.49	0.74
HADS	1.16	0.33	0.59	0.67
MMSE	1.32	0.27	2.36	0.57
MoCa	4.49	**0.002**	3.81	**0.006**
